# Regional and Cellular Mapping of Sortilin Immunoreactivity in Adult Human Brain

**DOI:** 10.3389/fnana.2019.00031

**Published:** 2019-03-12

**Authors:** Shu-Yin Xu, Qi-Lei Zhang, Qi Zhang, Lily Wan, Juan Jiang, Tian Tu, Jim Manavis, Aihua Pan, Yan Cai, Xiao-Xin Yan

**Affiliations:** ^1^Department of Anatomy and Neurobiology, Xiangya School of Medicine, Central South University, Changsha, China; ^2^SA Pathology, Schools of Medicine and Veterinary Science, Hanson Institute Centre for Neurological Diseases, The University of Adelaide, Adelaide, SA, Australia; ^3^Center for Morphological Sciences, School of Basic Medicine, Central South University, Changsha, China; ^4^Department of Histology and Embryology, Xiangya School of Medicine, Central South University, Changsha, China; ^5^Key Laboratory of Hunan Province in Neurodegenerative Disorders, Xiangya Hospital, Central South University, Changsha, China

**Keywords:** dementia, neuronal mapping, neurodegenerative diseases, neuropeptides, protein trafficking, Vps10p

## Abstract

Sortilin is a member of the vacuolar protein sorting 10 protein (VPS10P) domain receptor family, which carries out signal transduction and protein transport in cells. Sortilin serves as the third, G-protein uncoupled, receptor of neurotensin that can modulate various brain functions. More recent data indicate an involvement of sortilin in mood disorders, dementia and Alzheimer-type neuropathology. However, data regarding the normal pattern of regional and cellular expression of sortilin in the human brain are not available to date. Using postmortem adult human brains free of neuropathology, the current study determined sortilin immunoreactivity (IR) across the entire brain. Sortilin IR was broadly present in the cerebrum and subcortical structures, localizing to neurons in the somatodendritic compartment, but not to glial cells. In the cerebrum, sortilin IR exhibited differential regional and laminar patterns, with pyramidal, multipolar and polymorphic neurons in cortical layers II–VI, hippocampal formation and amygdaloid complex more distinctly labeled relative to GABAergic interneurons. In the striatum and thalamus, numerous small-to-medium sized neurons showed light IR, with a small group of large sized neurons heavily labeled. In the midbrain and brainstem, sortilin IR was distinct in neurons at the relay centers of descending and ascending neuroanatomical pathways. Dopaminergic neurons in the substantia nigra, cholinergic neurons in the basal nuclei of Meynert and noradrenergic neurons in the locus coeruleus co-expressed strong sortilin IR in double immunofluorescence. In comparison, sortilin IR was weak in the olfactory bulb and cerebellar cortex, with the mitral and Purkinje cells barely visualized. A quantitative analysis was carried out in the lateral, basolateral, and basomedial nuclei of the amygdaloid complex, as well as cortical layers II–VI, which established a positive correlation between the somal size and the intensity of sortilin IR among labeled neurons. Together, the present study demonstrates a predominantly neuronal expression of sortilin in the human brain with substantial regional and cell-type variability. The enriched expression of sortilin in pyramidal, dopaminergic, noradrenergic and cholinergic neurons suggests that this protein may be particularly required for signal transduction, protein trafficking and metabolic homeostasis in populations of relatively large-sized projective neurons.

## Introduction

Sortilin was originally purified via receptor-associated affinity chromatography from human brain extracts and characterized as a type I transmembrane protein belonging to the vacuolar protein sorting 10 protein (VPS10P) domain receptor family (Petersen et al., 1997). Many signaling proteins have been identified as sortilin ligands in the central and peripheral systems (Mazella et al., 1998; Nykjaer and Willnow, 2012; Quistgaard et al., 2014; Xu et al., 2018). Thus, sortilin serves as an ApoE receptor on neuronal and non-neuronal cells, which may involve in the development of some cardiovascular, metabolic and neurological diseases (Carlo et al., 2013; Coutinho et al., 2013; Kjolby et al., 2015; Strong, 2018). Sortilin can bind to nerve growth factors especially their preforms, thereby modulating neuronal viability and function critical for brain health and disease (Nykjaer et al., 2004; Chen et al., 2005; Arnett et al., 2007; Capsoni et al., 2013). Sortilin may involve in mood disorders (Buttenschøn et al., 2015; Devader et al., 2017; Moreno et al., 2018), and in brain aging and Alzheimer’s disease (AD) pathogenesis by interplaying with β-amyloid precursor protein (APP), β-secretase-1 (BACE1) and tau (Finan et al., 2011; Gustafsen et al., 2013; Yang et al., 2013; Andersson et al., 2016; Johnson et al., 2017; Xu et al., 2018). In fact, sortilin can form novel C-terminal fragments to deposit as senile plaque-like lesions in aged and AD human brains (Hu et al., 2017; Zhou F.Q. et al., 2018). Genetic and pathological evidence also emerges pointing to a link of sortilin to frontotemporal dementia, possibly via its interaction with progranulin (Carrasquillo et al., 2010; Hu et al., 2010; Prudencio et al., 2012; Philtjens et al., 2018). Moreover, sortilin may sort/transport protein products into the lysosomal system, which is key to intracellular protein homeostasis but might be relevant to the occurrence of intraneuronal proteinopathies (Canuel et al., 2009; Dumaresq-Doiron et al., 2013; Zhou et al., 2015; Paushter et al., 2018; Zhou X. et al., 2018).

One initially identified role for sortilin involves its signal transduction for neurotensin (NT), as the third, non-G-protein coupled receptor, i.e., NT receptor-3 (NTR3) (Mazella et al., 1998; Sarret et al., 2003a; Quistgaard et al., 2009, 2014). NT is a small peptide consisted of thirteen amino-acid residues (Carraway and Leeman, 1973). Pharmacological studies utilizing NT and/or its agonists/antagonists reveal a wide range of neurotransmitter-like effects of this peptide in the central and peripheral nervous systems (Gully et al., 1993; Alonso et al., 1999; Tyler-McMahon et al., 2000; Hong et al., 2002; McCormick and Stoessl, 2003; Ogawa et al., 2005). These effects are likely mediated by the G-protein coupled neurotensin receptors 1 and 2 (NTR1 and NTR2), and sortilin/NTR3, in the nervous system (Kitabgi et al., 1977; Lazarus et al., 1977; Uhl et al., 1977; Manberg et al., 1982; Sadoul et al., 1984; Köhler et al., 1987; Moyse et al., 1987; Waters et al., 1987; Nicot et al., 1994; Boudin et al., 1996; Chalon et al., 1996; Lahti et al., 1998; Fassio et al., 2000; Sarret et al., 2003a,b). Overall, NT has been shown to regulate the activity and function of multiple neuronal domains, including the glutamatergic (Chen et al., 2006; Ferraro et al., 2011); dopaminergic (Palacios and Kuhar, 1981; Faggin et al., 1990), noradrenergic (Olpe and Steinmann, 1991), cholinergic (Szigethy and Beaudet, 1987) and serotonergic (Li et al., 2001) systems in the brain, as well as the peptidergic neurons in the gut (Helmstaedter et al., 1977; Polak and Bloom, 1979). Other studies have demonstrated that abnormal NT signaling could be related to the pathophysiology of various neurological and psychiatric disorders including Parkinson’s disease (Uhl et al., 1984; Chinaglia et al., 1990; Fernandez et al., 1996), AD and dementia (Benzing et al., 1990), pain (Feng et al., 2015), depression and schizophrenia (Cervo et al., 1992; De Wied and Sigling, 2002; Hamid et al., 2002; Boules et al., 2014). Therefore, NT agonists/antagonists including that acting on sortilin/NTR3 have been considered for pharmaceutical applications (Kitabgi, 2002; Kinkead and Nemeroff, 2006; Xiao et al., 2014; Griebel, 2015).

A detailed regional/cellular map of normal sortilin expression in adult human brain would be informative for further understanding of its normal and abnormal neurobiological functions through mediating receptor-ligand interaction or intracellular protein sorting/trafficking. The current mapping study was thus carried out using a group of postmortem brains from mid-age to aged subjects free of neuropathology, with sections from representative structures/regions processed immunohistochemically using specificity-verified sortilin antibodies (Hu et al., 2017). We also attempted to address the particularly strong sortilin expression among some subsets of neurons via morphometric analysis and double immunofluorescence.

## Materials and Methods

### Human Brain Samples

Postmortem human brains were banked through the willed body donation program at Xiangya School of Medicine using a Standardized Operational Protocol (SOP) established by the China Human Brain Banking Consortium, which includes a signed antemortem consent from the donor and family to donate body/organs after death for medical education and research (Yan et al., 2015; Qiu et al., 2018). Use of postmortem human brains was approved by the Institutional Committee for Research and Education, in compliance with the code of ethics of the World Medical Association (Declaration of Helsinki) and guidelines developed by the National Institutes of Health.

The current study used formalin-fixed half brains from selected donors died of non-neurological diseases, with the demographic information of the cases (age at death, gender, disease cause of death, and postmortem delay of brain collection) listed in Table 1. As a part of the screening histological evaluation in the SOP of human brain banking (Qiu et al., 2018), brain samples from all cases had been assessed immunohistochemically for β-amyloid (Aβ), phosphorylated tau (p-Tau), α-synuclein and transactive response DNA binding protein 43 kDa (TDP-43) in paraffin or cryostat sections from the temporal, parietal, frontal and occipital lobes. The cases were selected to use in the current study on the basis that none of the above neuropathologies were found in the examined brain regions.

**Table 1:** Demographic information of brain donors.

Case number	Age at death (years)	Gender	Cause of death	Postmortem delay (hours)
#1	35	M	Liver cancer	7
#2	50	F	Leukemia	8
#3	55	M	Liver cancer	8
#4	59	M	Colon cancer	12
#5	60	M	Heart failure	14
#6	70	M	Lung cancer	8

### Tissue Processing

After removal from the skull, each brain was bisected along the cerebral sagittal fissure, with one hemi-brain immersed in phosphate-buffered formalin for histological studies and the other hemi-brain fresh-frozen for future biochemical studies (after cut into frontal slices in 1 cm thickness). After formalin immersion for 2–4 weeks, the fixed half brain was sliced frontally at 1 cm thickness, with the olfactory bulb (OB) and tissue blocks containing, or at the levels of, the frontal, parietal and occipital lobes, striatum, thalamus, cerebellar cortex and deep nuclei, middle brain, pons, and medulla oblongata embedded with paraffin or placed in 10 to 30% sucrose in 0.01 M phosphate buffer (see Supplementary Figure 1). Brain tissues were then prepared into paraffin (5 μm thick) or cryostat (40 μm thick) sections. The cryostat sections were first collected in phosphate-buffered saline (PBS, 0.01 M, pH 7.2) after cutting, then rinsed with PBS twice to remove the embedding medium, and finally stored in a cryoprotectant at -20°C before use for immunolabeling. During cutting, adjacent sections from the same brain block were collected alternatively into 24 sets of wells of cell/tissue culture plates, such that each well contained 4 to 6 equally spaced (24 × 40 μm ≈ 1000 μm in distance) sections. Thus, multiple sets of adjacent sections could be obtained from neighboring wells in case of specific experimental examinations, for instance mirror-section comparative studies on different antibody labelings.

### Immunohistochemistry

Sections from multiple regions and cases were immunohistochemically processed in parallel in each experiment. Thus, sets of sections were consistently stained with the goat antibody raised against sortilin extracellular domain (diluted at 1:2000, Cat#AF3154, raised against recombinant Gly76 to Asn753 of human sortilin, R&D Systems China Co., Ltd., Shanghai, China) and the rabbit antibody targeting sortilin intracellular C-terminal domain (1:2000, Cat#ab16640, raised against recombinant 800–831 a.a. of human sortilin, Abcam Trading Shanghai Company Ltd., Shanghai, China). The specificity of these two antibodies was thoroughly verified by immunoblotting and immunohistochemical methods using sortilin knockout mouse brains as well as antibody omission and absorption assays with human brain sections (Hu et al., 2017). We further verified the specificity of the two primary antibodies using prefrontal and cerebellar cortical sections in this study (Supplementary Figure 2).

Immunohistochemical labeling was initiated with treatments of free-floating sections in PBS containing 5% H_2_O_2_ for 30 min and containing 5% normal horse serum with 0.3% Triton X-100 for 1 h. Sections were subsequently incubated in PBS containing the sortilin antibodies at 4°C overnight. After several washes with PBS, sections were reacted with biotinylated pan-specific secondary antibody (horse anti-mouse, rabbit and goat IgGs) at 1:400 for 1 h and avidin-biotin complex (ABC) reagents (1:400) (Vector Laboratories, Burlingame, CA, United States) for another hr. Immunoreactive product was visualized with 0.003% H_2_O_2_ and 0.05% 3,3′-diaminobenzidine (DAB). After stopping the DAB reaction with further rinses in PBS, the immunolabeled sections were mounted on glass microslides, dehydrated and coverslippered. One or two sections per brain block/region were also counterstained with hematoxylin before dehydration. In addition, several cortical and subcortical sections were included in each staining experiment except for the step of primary antibody incubation. These sections were thus processed with the omission of the primary antibody, and they were used for measuring background labeling intensity in the event of densitometric analysis.

### Double Immunofluorescence

Double immunofluorescence was used to explore the neurochemical phenotypes of sortilin-labeled neurons in representative brain structures. Thus, temporal neocortical and cerebellar sections were processed for double labeling for sortilin with selective neuronal and glial markers, while basal forebrain and brainstem sections were processed for the colocalization of sortilin in dopaminergic, cholinergic and norepinephrinergic neurons. Double labeling was initiated with a treatment of sections in PBS containing 5% donkey serum for 30 min. The sections were then incubated overnight at 4°C with the following pairs of primary antibodies: (1) goat anti-sortilin (AF3154, 1:1000, same below) and mouse anti-neuron-specific nuclear antigen (NeuN, 1:4000, MAB377; Millipore, Temecula, CA, United States); (2) goat anti-sortilin and mouse anti-calbindin (CB, C9848, 1:4000; Sigma-Aldrich, St Louis, MO, United States); (3) goat anti-sortilin and mouse anti-parvalbumin (PV, 1:4000, P3088, Sigma-Aldrich); (4) goat anti-sortilin and mouse anti-tyrosine hydroxylase (TH, T2928, 1:4000, Sigma-Aldrich); (5) goat anti-sortilin and mouse anti-glial fibrillary acidic protein (GFAP, 1:2000; MAB360, Millipore); (6) rabbit anti-sortilin (ab16640, 1:1000) and goat anti-Sp8 (sc-104661, 1:1000, Santa Cruz Biotech, Inc., United States) (Zhang et al., 2016); (7) rabbit anti-sortilin and goat anti-choline acetyltransferase (AChE) (AB1447, 1:4000; Millipore), (8) rabbit anti-sortilin and goat anti-Iba1 (ab5076, 1:4000; Abcam). Sections were then incubated at room temperature for 2 h with Alexa Fluor^®^ 488 and Alexa Fluor^®^ 594 conjugated donkey anti-mouse, anti-rabbit or anti-goat IgG (1:200, Invitrogen, Carlsbad, CA, United States). After several rinses with PBS, the sections were mounted on glass microslides, counterstained with bisbenzimide (Bis), treated with 0.1% Sudan black to block autofluorescence, and coverslippered with an anti-fading medium before microscopic examination.

### Imaging, Quantitative Analysis, Statistical Test and Figure Preparation

Immunolabeled sections were examined on an Olympus BX51 microscope (CellSens Standard, Olympus Corporation, Japan) for initial analysis of labeling quality. For each region/structure of a given brain, sections at 2–3 levels representing the characteristic regional and cellular pattern of sortilin labeling were scan-imaged using the 20× objective on a Motic-Olympus microscope equipped with an automated stage and imaging system (Wuhan, Hubei, China), which could yield a final auto-focused, montaged, and magnification-adjustable image covering the entire area of a glass slide. Low (1×) and high (10× and 20×) magnification images over the area of interest were then prepared from the montaged images for each brain region/structure for figure illustration. These images were also used for quantitative analyses, with somal size and optic density measurements carried out for selected groups of sortilin labeled neurons using the OptiQuant software (Packard Instruments, Meriden, CT, United States). The methodological details will be provided in the result sections along with specific illustrations. Immunofluorescent images were captured on a Nikon confocal microscope (Nikon Instruments, Inc., Japan) using 20× or 40× objectives through 3–5 scans covering a total tissue depth <10 μm. Single-cannel and merged images were obtained using the Nikon-EZ-C1 3.70 Free-Viewer software. Quantification of colocalization rates (sortilin vs. neuronal markers) was carried using 20× immunofluorescent images from three brains (cases #3, 4, 5, and 6), which were captured over the temporal neocortical layers and hippocampal CA1 region (sampling methodology to be detailed in the result section). The non-parametric test (Kruskal–Wallis analysis with Dunn’s multiple) was used for comparison of medians between groups. Pearson correlation was used for statistical analyses of quantified data of somal size and optic density (GraphPad Prism 5, San Diego, CA, United States). Figure panels were assembled using Photoshop 7.1.

## Results

We obtained optimal immunolabeling with both the goat and rabbit sortilin antibodies in the processed cryostat sections from all cases. We also used paraffin sections from a few cases to verify the laminar and cellular pattern of sortilin IR, with examples of micrographs shown for comparison. Consistent with earlier observations (Hu et al., 2017; Zhou F.Q. et al., 2018), the two antibodies exhibited essentially identical labeling pattern with the IR localized to neuronal profiles in all brains examined. Therefore, for simplicity, we will first denote the regional and cellular expression pattern of sortilin based on the peroxidase-DAB immunolabeling with the goat antibody only, using a complete set of images from a mid-age case (case #5). For a given neuroanatomical region, low magnification figures will demonstrate the overall distribution of the labeling, while high magnification illustrations depict the detailed morphology of the labeled cells. We further report a correlation between somal size and immunolabeling intensity among labeled neurons in the amygdala and cerebral cortex based on quantitative analyses. Lastly, we show confocal double immunofluorescent characterization of sortilin-labeled cells in representative brain regions. For this set of data, immunofluorescent images derived from labeling with both the goat and the rabbit sortilin antibodies are demonstrated. Neuroanatomical structures were marked in reference to human brain atlases (Brodmann, 1909; Mai et al., 2008; Ding et al., 2017).

### Sortilin IR in Major Neuroanatomical Structures of the Human Brain

#### Olfactory Bulb

**Figure 1:**
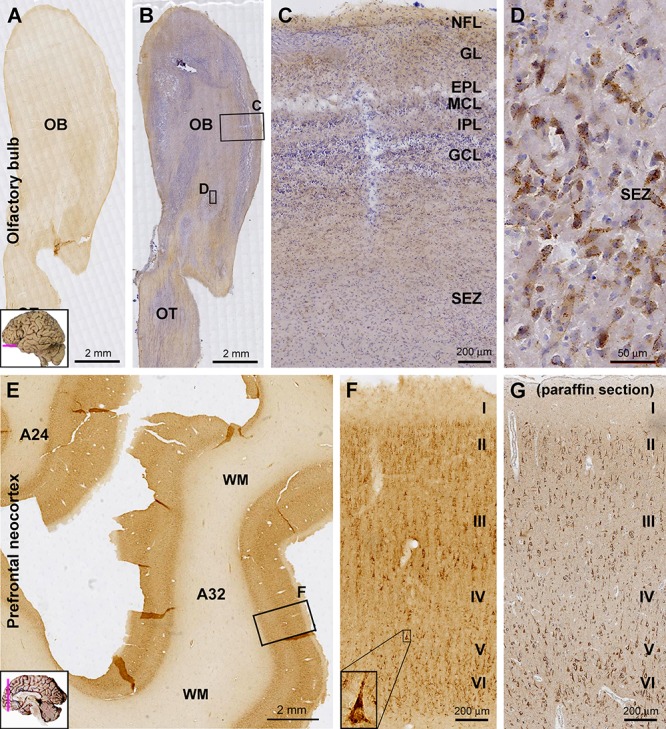
Laminar distribution and cellular localization of sortilin immunoreactivity (IR) in adult human olfactory bulb (OB) and prefrontal cortex. Panels **(A,B)** are low power views of sortilin IR in the bulb, with the latter taken from a hematoxylin-counterstained section. Panels **(C,D)** are enlarged views from the framed areas in **(B)** as indicated. The IR is generally weak across the various layers of the bulb **(C)**, except for some lightly labeled fusiform cells around the central bulb area **(D)**, likely representing the subependymal zone (SEZ). Panel **(E)** shows a low magnification view of labeling in the prefrontal cortex. The pattern is the same for Brodmann areas 32 and 24, with the IR occurring in the cortex but little in the white mater (WM). Panel **(F)** is enlarged from the framed area in **(E)** as indicated, showing labeled cells present largely in layers II–III and V/VI, primarily pyramidal and polymorphic in morphology. A large-sized pyramidal neuron in layer V exhibits the darkest labeling and contains intracellular granules (**F**, insert). Panel **(G)** shows an example of sortilin IR revealed in paraffin section of the prefrontal cortex (with hematoxylin-counterstain); the laminar and cellular pattern of the labeling are comparable to that displayed in cryostat sections **(F)**. A small human brain map is inserted in the low magnification panels to indicate the tissue sampling locations and section orientation (provided in other figures as well). OT, olfactory tract; NFL, nerve fiber layer; GL, glomerular layer; EPL, external plexiform layer; MCL, mitral cell layer; IPL, inner plexiform layer; GCL, granule cell layer. I–VI, cortical layers. Scale bars are as indicated.

At low magnification, the overall intensity of sortilin IR was low across the OB in longitudinal sections covering the bulb and olfactory tract (OT), with no distinct laminar pattern identifiable (Figure 1A). In sections counterstained with hematoxylin, the laminar architecture of the bulb was displayed in the background, with diffuse light brown IR seen between hematoxylin-labeled (blue) nuclei (Figure 1B). No immunolabeled cellular profiles could be reliably identified from the nerve fiber layer (NFL), glomerular layer (GL), mitral cell layer (MCL), and granule cell layer (GCL) (Figure 1C). Some lightly stained cells were seen around center of the bulb, representing the subependymal zone (SEZ) (Figure 1B). These cells were largely fusiform in shape and contained intracellular granules (Figure 1D).

#### Cerebral Cortex

In general, sortilin IR was fairly intense throughout neocortical areas of the cerebrum. The IR was located primarily in the gray matter, while layer I exhibited much lighter reactivity, only slightly above the level (regarded as background) seen in the white matter (WM). Sortilin IR was distributed with clear laminar patterns that could be related to the characteristic laminar architectures of individual functional cortical areas. Moreover, based on close microscopic examination, sortilin IR could be largely interpreted to be primarily associated with pyramidal, multipolar and polymorphic neurons, i.e., largely the projective neuronal populations of the cortex (Figure 1E–G, 2–4, 5A).

Across the prefrontal neocortex, sortilin IR exhibited a similar laminar distribution pattern in various subregions such as Brodmann area 24 and area 32 (Figure 1E). Labeled cells were mainly seen in layers II–III, V, and VI, mostly appeared as small to medium-sized pyramidal and polymorphic neurons. Many large-sized cells at the low part of layer III were strongly labeled and appeared typically pyramidal in shape (Figure 1F,G). A few large-sized pyramidal neurons in layer V also showed heavy IR in the somata, apical and basal dendrites, while no axonal processes were seen around the basal part of perikarya (Figure 1F, insert). Shown as an example, the laminar and cellular labeling pattern of sortilin IR seen in the paraffin sections of the cerebral cortex was comparable to that revealed in the cryostat sections (Figure 1F,G). However, there lacked a differential lamination pattern of the neuropil labeling in paraffin sections in comparison to cryostat sections, which was due to the much reduced tissue thickness (5 vs. 40 μm) in the former relative to the later preparations.

**Figure 2:**
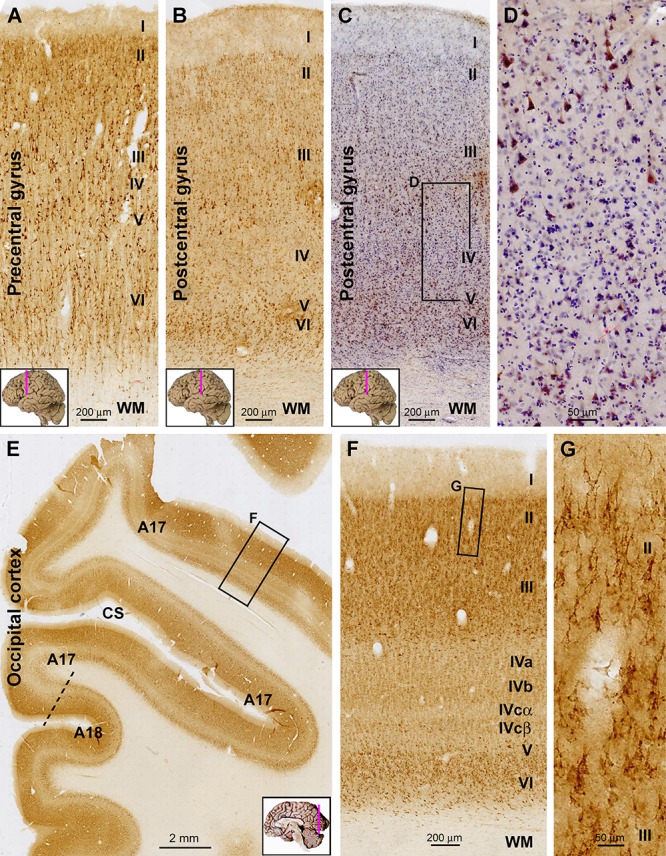
Representative images illustrating sortilin IR in the primary motor (precentral gyrus), somatosensory (postcentral gyrus), and visual cortices of adult human brain. Cortical regions and lamination are as indicated in individual panels. Note the narrow layer IV **(A)** and V **(B)** in the motor and somatosensory areas, respectively. In the hematoxylin-counterstained sections, large pyramidal neurons with heavy IR are clearly present in the low portion of layer III, whereas layer IV has few labeled cells **(C,D)**. In the occipital cortex, the border between areas 17 and 18 can be identified based on the widening and stratification of layer IV in the former **(E)**. Again, sortilin IR occurs mainly in layers II/III and VI **(F)**, with those in layers II/III localized to pyramidal neurons **(G)**. Note that the layer V of area 17 is thin, whereas layer IV is expanded and can be divided into IVa, IVb, IVcα, and IVcβ based on the difference in labeling intensity **(F)**. CS, calcarine sulcus. Scale bars are as indicated.

Sortilin IR in the primary motor (Brodmann areas 4, 6) and primary somatosensory (areas 3, 1, 2) cortices well depicted the featured laminar architectures in these functional areas, i.e., minimal depths of layers IV and V in the former and later, respectively (Figure 2A–C). In the motor cortex, large-sized pyramidal cells in low layer III and layer V (potentially Betz cells) showed strong IR, whereas moderate IR was seen in layer II to upper III and layer VI (Figure 2A). In the somatosensory cortex, the wide layer IV exhibited weak IR relative to other cortical mantle layers. Again, a population of large-sized pyramidal cells in deep layer III were heavily labeled, clearly seen in sections with hematoxylin counterstain (Figure 2C,D).

In immunolabeled sections from the occipital lobe, the border between areas 17 and 18 could be clearly identified based on the transition in the appearance of layer IV (Figure 2E). In the primary visual cortex (area 17), the entire layer IV exhibited lower labeling intensity relative to layers II–III and V–VI. Although it was fairly difficult to identify individual labeled cells over layer IV, sortilin IR visualized its sublaminar organization as layers IVa, b, and c based on a differential labeling neuropil intensity. Further, layer IVcα appeared as a lighter band relative to IVcβ, visible along the gyral and sucal regions of area 17 (Figure 2E,F). As with the aforementioned neocortical areas, the morphology of labeled cells in layers II–III appeared pyramidal-like (Figure 2G). Different from area 17, the laminar pattern of IR in area 18 was similar to that seen in the prefrontal and temporal neocortex (i.e., without a sub-laminated layer IV) (Figure 2E), representing a pattern common to association neocortical regions.

**Figure 3:**
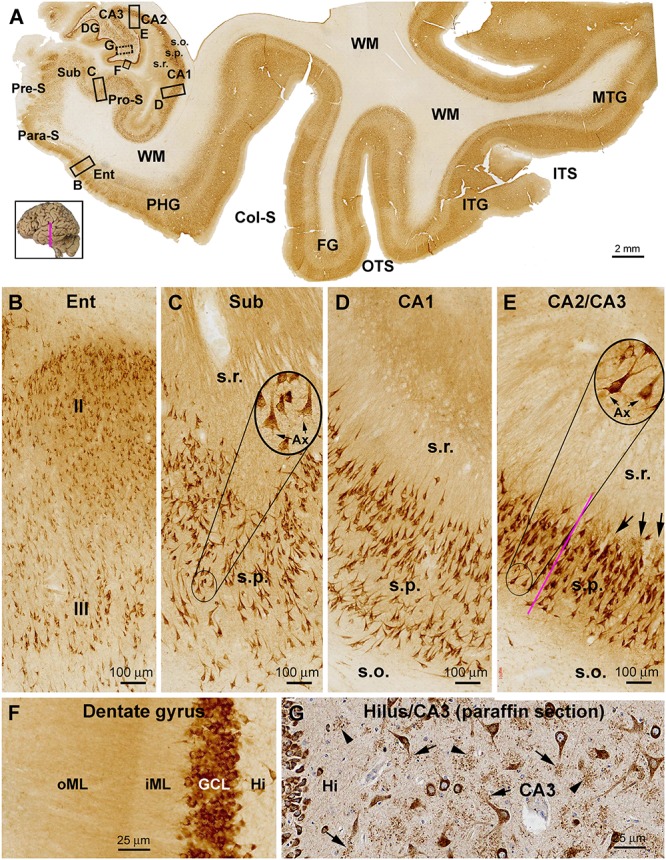
Representative images showing sortilin IR in the adult human temporal lobe structures. Panel **(A)** is a low magnification image from a frontal plane section passing the anterior part of the hippocampus. The laminar pattern of IR in the temporal neocortex is similar by moving lateromedially from the middle (MTG) to inferior (STG) temporal gyri, and further to the fusiform gyrus (FG), with a light band corresponding to layer IV seen across the areas. A transition of the six-layered neocortex to three-layered paleocortex is seen around the parahippocampal gyrus (PHG). Sortilin IR clearly displayed the stratum pyramidale (s.p.) over the subicular subregions including the parasubiculum (Para-S), presubiculum (Pre-S), subiculum (Sub) and prosubiculum (Pro-S), as well as subsectors of Ammon’s horn CA1, CA2, and CA3. Framed areas are shown as enlarged views of the labeling the areas as indicated **(B–G)**. Panel **(B)** shows labeled pyramidal-like neurons in layers II and III, with that in layer II arranged as a cell island. Panel **(C)** show labeled neuronal somata and dendritic processes in the subiculum in the s.p. and stratum radiatum (s.r.). Panel **(D)** shows labeled CA1 pyramidal neurons and dendritic processes. Panel **(E)** shows the labeling at the border (purple line) of CA2 and CA3, note the clusters of granular profiles representing thorny excrescences (TE) (arrows) in the CA3. Axons (Ax) of the pyramidal neurons are rarely visible or only traceable for a short distance from the somata (**C,E**, inserts). Panel **(F)** shows the dense labeling in the granule cells and the neuropil-like labeling in the molecular layer (ML) appearing as a lightly stained band over the inner ML (iML) and a moderately stained band over the outer ML (oML). Panel **(G)** shows an additional example (also see Figure 1G) of sortilin IR revealed in paraffin sections (with hematoxylin-counterstain), illustrating the TEs of the mossy cells and CA3 pyramidale neurons. The TEs appear as granular profiles packed around the dendritic trees (arrows) or in isolated clusters (arrowheads). ITS, inferior temporal sulcus; OTS, occipitotemporal sulcus; Col-S, collateral sulcus; WM, white matter. Scale bars are provided in each panel.

**Figure 4:**
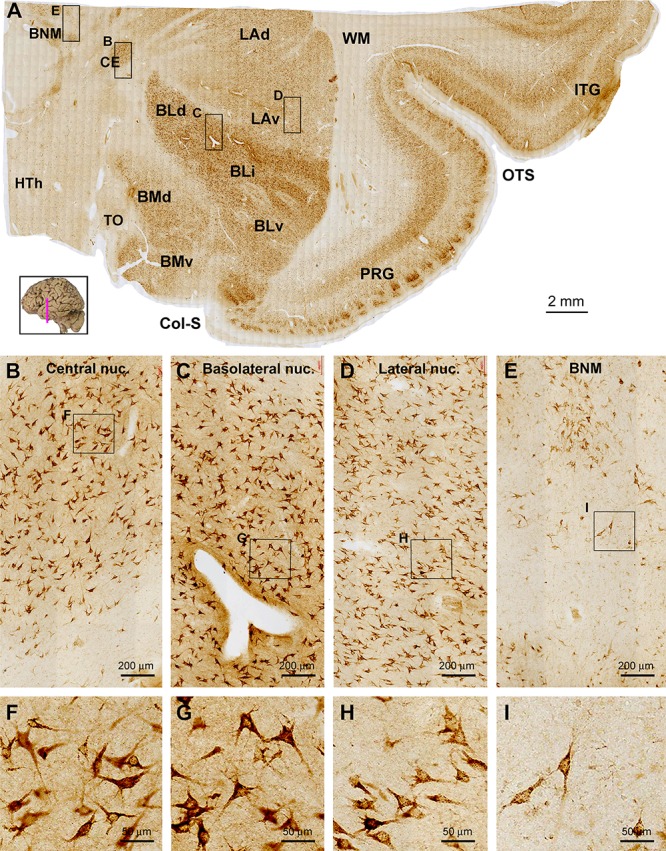
Images showing sortilin IR in the adult human amygdala complex and the surrounding temporal cortex. Panel **(A)** is a low magnification image covering the entire area, with framed areas enlarged as other panels as indicated. Across the amygdaloid complex, the central nucleus (CE) and basolateral nucleus (BL) show a darker labeling than the lateral nucleus (LA) and basomedial nucleus (BM) **(A–D)**. At high magnification, the labeled neurons are mostly multipolar in shape, appear to have similar soma size and are oriented without regularity in all subnuclei. The neurons in the CE and dorsal (BLd), intermediate (BLi) and ventral (BLv) divisions of the BL appear to be more strongly labeled relative to those in dorsal (LAd) and ventral (LAv) divisions of the LA and in the dorsal (BMd) and ventral (BMv) divisions of the BM (**B–D**, **F–H**). Medial to the amygdalar complex, labeled cells occurs in groups corresponding to the basal nucleus of Meynert (BNM) **(A,E,I)**, while a small amount of labeled cells with light to moderate intensity are seen in the hypothalamic region (HTh) **(A)**. Little labeling is seen in the what matter (WM) and the optic tract (OT). Col-S, collateral sulcus; OTS, occipitotemporal sulcus; PRG, perirhinal gyrus. Scale bars are provided in each panel.

In the temporal lobe neocortex, the laminar pattern of sortilin IR in the superior (STG), middle (MTG), and inferior (ITG) temporal gyri was also similar to that seen in the prefrontal cortex, with strong IR occurred over layers II, III, V, and VI, but weak IR in layer IV (Figure 3A, 4A). The laminar distribution pattern changed around the parahippocampal gyrus (PHG) as the cortex transited from the six-layered neocortex to three-layered paleocortex (Figure 3A). In the entorhinal cortex (Ent), layer II and upper III contained a large amount of clearly labeled neurons, with those in the former arranged as cell islands (Figure 3A,B). At the plane of the amygdalar complex, these cell islands emerged as moving from the ITG to the perirhinal gyrus (PRG) (Figure 4A). Many of these cell islands were further divided into a superficial part approximately in layer II and a deep part in upper layer III by a cell-spared zone (Figure 4A). In the subicular subregions including the pro-subiculum (Pro-S), subiculum (Sub), pre-subiculum (Pre-S), and para-subiculum (Para-S), sortilin labeled cells were essentially located in the stratum pyramidale (s.p.), with island arrangement seen in the superficial part of this layer as well (Figure 3A,B). At high magnification, the labeled cells appeared essentially multipolar or pyramidal in shape, with IR localized to the somata, apical and basal dendrites. The apical dendrites could be traced for a fairly long distance up to several times of the long axis of the somata, which also gave rise to a diffuse and moderate neuropil labeling in stratum radiatum (s.r.). In contrast, the axons of these neurons were either not labeled, or were traceable only for a very short distance from cell body (Figure 3A,C). There was relatively strong neuropil-like IR between the distinctly labeled somata in the layer II cell islands and subicular gray matter, likely representing transected dendritic elements (Figure 3B,C).

#### Hippocampal Formation

In the Ammon’s horn (CA1-3), heavy sortilin IR was localized to the stratum pyramidale (s.p.) (Figure 3A). Closer examination indicated that the somata and dendritic processes of CA1-3 pyramidal neurons were well labeled, with the latter extended over the s.p. and into the s.r. (Figure 3D,E). In CA3, clusters of immunolabeled granular profiles (pointed by arrows) were observed around the border of the s.p. and s.r. as well as between the somata and apical dendrites of pyramidal neurons, while these profiles were not present in the same lamina of CA2 (Figure 3D). These profiles were identified as the complex spine formation, i.e., thorny excrescences (TEs), on the dendrites of the CA3 pyramidal neurons (Hu et al., 2017).

In the dentate gyrus (DG), the labeling over the GCL was heavy, with the IR localized to individual granule cells (Figure 3A,F). The molecular layer (ML) exhibited fairly intense labeling apparently greater than that in the cortical WM. It should be noted that the labeling intensity in the inner 1/3 of the ML, or the iML, was lighter than that in the our 2/3 of the this layer, i.e., oML (Figure 3F). Large multipolar and fusiform cells in the hilus likely representing mossy cells were also strongly labeled, as were the pyramidal cells in the hilar region of the CA3 subsector. In addition, both the hilar mossy cells and CA3 pyramidal neurons possessed TEs on their dendrites, which could be identified in immunolabeled cryostat (not shown, see Hu et al., 2017) and paraffin sections (Figure 3G).

#### Amygdaloid Complex

The entire amygdalar nuclear complex exhibited fairly strong sortilin IR with its individual subnuclei distinguishable (Figure 4A). At low magnification, the central nucleus (CE), basolateral nuclear group including the dorsal (BLd), intermediate (BLi) and ventral (BLv) subdivisions showed noticeably stronger IR relative to the lateral nuclear (LA) and basomedial (BM) nuclear groups. The above regional difference in labeling intensity was due to moderate to heavy sortilin IR of individual neuronal perikarya in the above corresponding subregions (Figure 4B–D). At high magnification, the immunolabeled perikarya in all of the amygdalar nuclei appeared essentially triangular or multipolar in morphology, with their proximal dendrites clearly marked. The labeled neurons also contained fine granules in the somata and dendritic processes (Figure 4F–H). In the same sections at the amygdalar levels, neuronal cells in the basal nuclei of Meynert (BNM) located mediodorsal to the amygdaloid complex were visualized (Figure 4A). The labeled cells in the BNM were largely multipolar and fusiform in shape, and often had long dendritic processes oriented to different directions. Large sized multipolar neurons appeared to consistently exhibit strong sortilin IR, whereas cells in smaller size displayed light labeling intensity (Figure 4E,I).

#### Basal Ganglia

**Figure 5:**
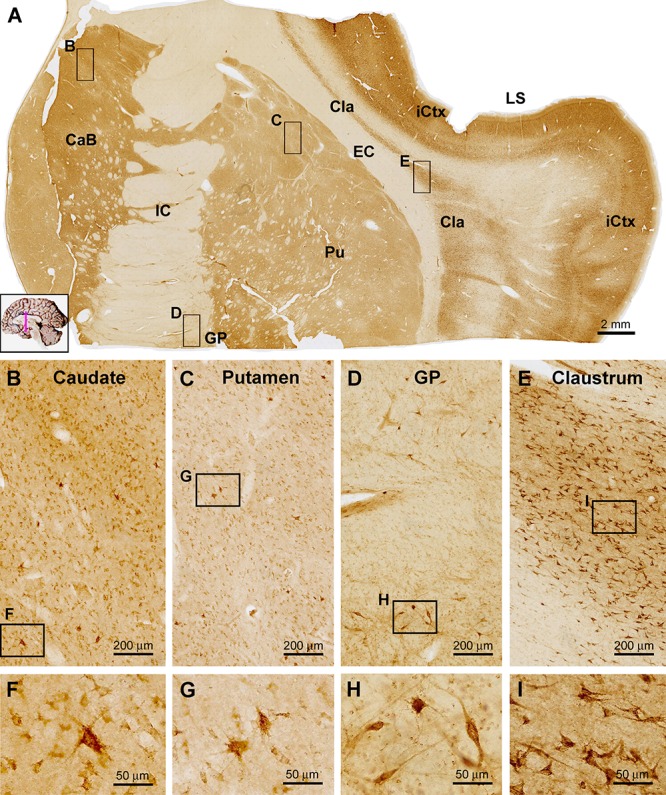
Images showing sortilin IR in subregions of the basal ganglia. Panel **(A)** is a low power view of frontal section at the level of the insular lobe, with framed areas enlarged as other panels as marked. Compared to the strong labeling in the insular cortex (iCtx) and the claustrum nucleus (Cla), light to moderate IR is seen in the body of caudate (CaB), the putamen (Pu) and globus pallidus (GP). At higher magnifications, the labeled cells in the above areas are mostly small multipolar neurons exhibiting light to moderate intensity. However, individual large-sized multipolar neurons are heavily labeled **(B,C,D,F,G,H)**. Panels **(E,I)** show high power views of the cells in the Cla, which exhibit stronger labeling intensity relative to the labeled neurons in the striatum. Note the lack of sortilin IR in the internal (IC) and external (EC) capsules. LS, lateral sulcus. Scale bars are as indicated.

**Figure 6:**
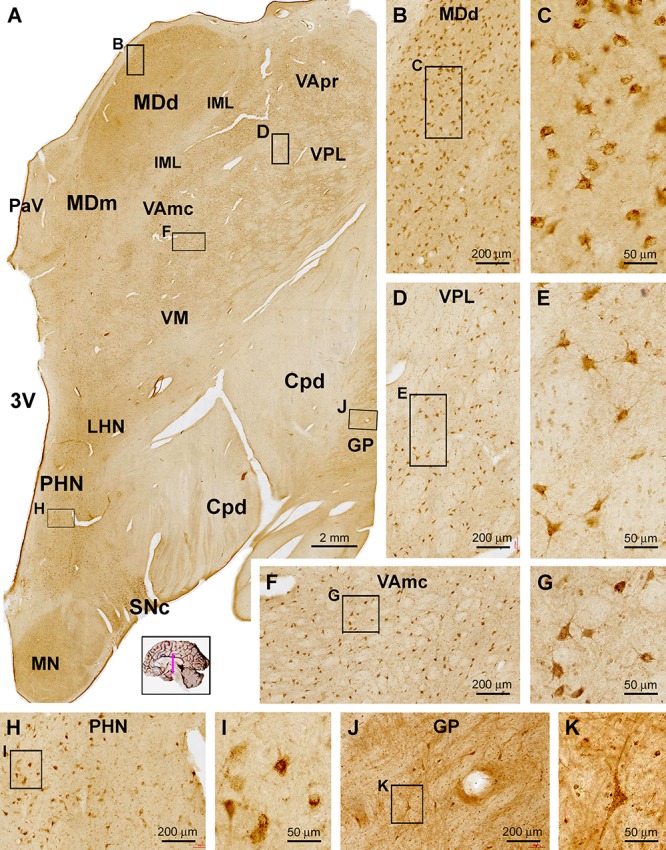
Distribution of sortilin IR in subregions of the thalamus and hypothalamus. Panel **(A)** is a low power view of a frontal section at the level of the mammillary body. The labeling intensity of sortilin IR is low in general across the thalamic and hypothalamic regions. Framed areas are enlarged as indicated to display immunolabeled cells in different locations. Panels **(B,C)** show small-sized multipolar neurons in the densocelllular division of the mediodorsal nuclear complex (MDd), which display moderate labeling intensity. Panels **(D–G)** show small-sized multipolar neurons with light labeling intensity in the lateral division of the ventroposterior nuclear complex (VPL), and in the magnocellular division of the ventral lateral nuclear complex (VAmc). Panels **(H,I)** show lightly stained neurons in posterior hypothalamic nucleus (PHN). In comparison, labeled neurons in the substantia nigra pars compacta (SNc) exhibit strong IR relative to the cells in the above thalamic and hypothalamic areas **(J,K)**. Cpd, cerebral peduncle; GP, globus pallidus; IML, internal medullary lamina; LHN, lateral hypothalamic nucleus; MDm, magnocellular (medial) division of the mediodorsal nucleus; PaV: paraventricular nucleus; 3V, third ventricle; VApr, parvocellular division of ventral lateral nucleus; VM, ventral medial nucleus. Scale bars in the panels are as indicated.

In frontal plane sections covering subregions of the stratum, internal capsule (IC) and thalamus, sortilin IR was seen in both the neostriatum and paleostriatum, whereas the IC exhibited no immunolabeling (Figure 5A). There also existed many unlabeled small areas in round and oval shapes inside the body of the caudate (CaB) as well as the putamen (Pu) and globus pallidus (GP), likely representing the penetrating bundles of neural tracts. The overall labeling intensity in the CaB appeared lighter as compared to that in the insular cortex (iCtx) as seen in the same section, but was noticeably stronger relative to the Pu and GP (Figure 5A). At high magnification, a great amount of small sized neuronal cells with weak sortilin IR were observed in the CaB and Pu (Figure 5B,C,F,G), mostly likely representing the so-called small spiny neurons (GABAergic) as characterized in human striatum. However, a few apparently large-sized multipolar neurons were heavily labeled in these regions, morphologically corresponding to the so-called large spiny neurons (cholinergic) in human striatum. It should be noted that the amount of labeled cells in the GP was much less relative to the CaB and Pu as examined at high magnifications (Figure 5A,D,H). The labeled GP neurons were mostly large in size with long-extending dendritic processes (Figure 5H, also see Figure 6A,J,K). Between the striatum and neocortex, the claustrum (Cla) exhibited fairly strong labeling intensity (Figure 5A). The immunolabeled neurons were mostly multipolar seen at high magnification, with their somata oriented tangentially to the insular cortex (Figure 5E,I).

#### Diencephalon

In general, the diencephalon parts of the brain exhibited lower labeling intensity relative to the cerebral structures. Thus, at low magnification sortilin IR was observed across the thalamic nuclear groups, which were separated by some unlabeled zones representing regions occupied by transected neural tracts (Figure 6A). Also, within a given nuclear region, small areas of unlabeled spots were distributed between groups of labeled cells. light to moderate reactivity was observed over mediodorsal nuclear complex including the densocelllular (MDd) and magnocellular (MDm) subdivisions, the ventral nuclear complex including parvocellular division (VApr), magnocellular division (VAmc) and the ventral medial nucleus (VM) (Figure 6A). At high magnification, the majority of the labeled cells showed weak to moderate labeling intensity, and were in multipolar shape with some in oval or fusiform shape (Figure 6B–G). There were also lightly stained neurons in multiple and fusiform ships in other thalamic and hypothalamic regions or nuclear groups, including the paraventricular area (PaV) of the thalamus, and anterior, posterior (PHN) and lateral nuclei (LHN) as well as the mammillary nuclear groups (MN) (Figure 6A,H,I). In comparison, in the superior end of substantia nigra pars compacta (SNc), there was a group of cells that exhibited strong IR (Figure 6A). Lateral to the unlabeled cerebral peduncle (Cpd), GP contained sparsely distributed neurons with relative large somal size long dendritic processes (Figure 6A,J,K).

#### Midbrain

**Figure 7:**
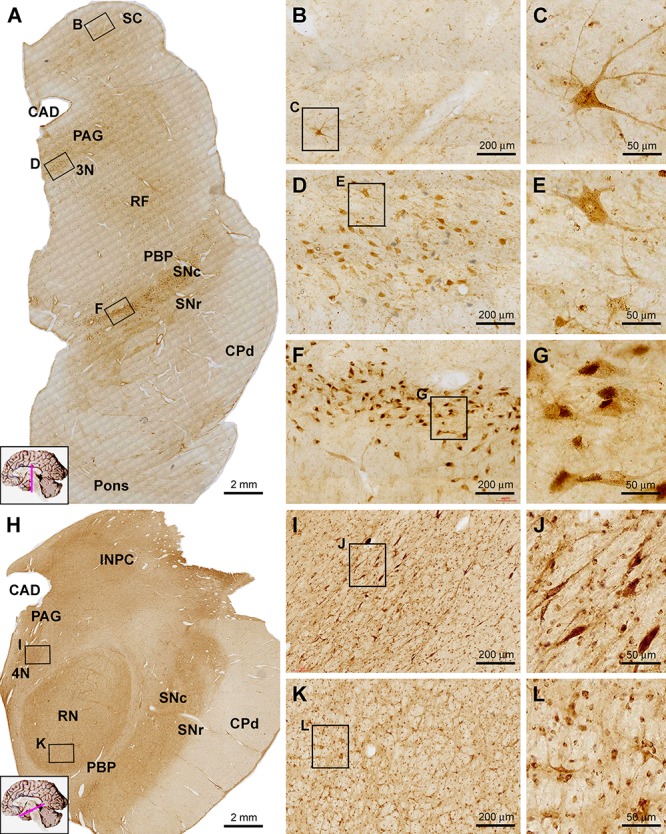
Images showing sortilin IR in the midbrain at the levels of superior colliculus **(A–G)** and red nucleus **(H–L)**. Panel **(A)** is low power view of labeling across the midbrain and part of pons, noting the prominent IR along the pars compacta of the substantia nigra (SNc) and moderate labeling located ventrally, laterally and dorsally to the cerebral aqueduct (CAD), corresponding to the periaqueductal gray substance (PAG), the oculomotor nucleus (3N) and reticular formation (RF). Panels **(B,C)** showed labeled cells in the deep layer of SC, including a large multipolar neuron. Panels **(D,E)** show moderately labeled neurons in oculomotor (3N) nucleus, some of which are multipolar. In the SNc, labeled cells are densely packed with heavy reactivity **(F,G)**. At the level of RN **(H)**, sortilin IR appears moderate across the medial and dorsal areas of the section, whereas no labeling exists in the cerebral peduncle (CPd) and less labeling occurs in the pars reticulata of SN (SNr) and parabrachial pigmented nucleus (PBP). Panels **(I–L)** show closer views of labeled cells in the trochlear nucleus (4N) and the red nucleus (RN). Scale bars are as indicated.

Sortilin IR was observed in selective locations in transverse sections of the midbrain at different neuroanatomical planes (Figure 7). In low power view of the section at the level of superior colliculus (SC), the immunolabeling appeared more impressive in the dorsal areas around the central cannel and a ventral area deep to the unlabeled cerebral peduncle (CPd) (Figure 7A). Sparsely distributed cellular profiles were found between the above two regions including the reticular formation (RF) (Figure 7A). At high magnification, lightly stained small-sized as well as a few moderately stained large-sized neurons were seen in the deep layer of the SC (Figure 7B,C). Many oval, fusiform and multipolar neuronal cells with moderate to strong staining intensity were observed in the periaqueductal gray substance (PAG) and the oculomotor nucleus (3N) (Figure 7A,D,E). Similarly, the labeling appeared light to moderate at low magnification across the mediodorsal regions in the distal part of midbrain (Figure 7H). However, individual neurons were clearly labeled in gray matter areas, such as the trochlear nucleus (4N), the interstitial nucleus of posterior commissure (INPC) and the red nucleus (RN) (Figure 7H–L). It should be noted that the labeling at the SNc appeared most impressive among the midbrain structures. Thus, a dense group of labeled cells were packed in SNc, which were in medium to large size, in round or oval shape, and often contained a dark brown pigment-like inclusion in the cytoplasm, likely representing the dopaminergic neurons predominately occupying over this location (Figure 7A,F,G).

#### Cerebellum

**Figure 8:**
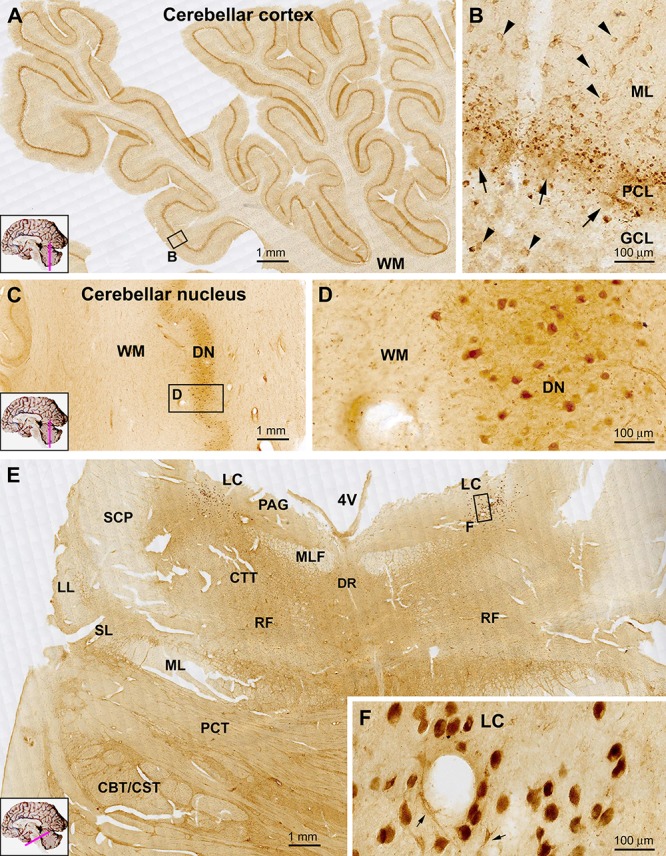
Representative images showing sortilin IR in the adult human cerebellum **(A–D)** and pons **(E)**. Panel **(A)** is low power view of the cerebellar cortex, with an impressive band-like labeling located in the middle of the cerebellar lobules. The framed area in **(A)** is enlarged as **(B)**, which shows that at high magnification, this band reflects small granular but non-cellular elements distributed along the Purkinje cell layer (PCL). The somata of Purkinje cells (pointed by arrows) exhibit faint labeling. Lightly stained small neurons (pointed by arrowheads) representing basket cells are present in the molecular layer (ML), with a few also in the granule cell layer (GCL). Cells in the dentate nucleus (DN) are lightly stained with their proximal processes visible. The cerebellar white matter (WM) shows no labeling. In transverse section of the upper part of the pons **(E)**, the most impressive sortilin IR occurs in the locus coeruleus (LC) that is occupied by a group of densely labeled neurons, as enlarged in **(F)**. These cells are mostly round/oval in shape and have short processes (insert). There are light labeling in the periaqueductal gray substance (PAG), dorsal raphe nucleus (DR) and reticular formation (RF). Little labeling is seen in the longitudinal and transverse neural fiber systems, including the central tegmental tract (CTT), median longitudinal fasciculus (MLF), superior cerebellar peduncle (SCP), lateral lemniscus (LL), spinal lemniscus (SL), medial lemniscus (ML), pontocerebellar tract (PCT) and the corticobulbar and corticospinal tracts (CBT, CST) **(E)**. Scale bars are as indicated.

At low magnification sortilin IR was seen in the cerebellar cortex with essentially no labeling in the WM (Figure 8A). The IR exhibited an impressive laminar pattern, with a narrow band-like immunolabeling at the middle part of the cortex along the cerebellar lobules. At high magnification, this band-like structure was located around the Purkinje cells layer (PCL). However, the labeled profiles were consisted of variably shaped elements in sizes much smaller than that of all neuronal cell types in the cerebellum. It should be noted that the very large somata of Purkinje cells were sometimes visible on the background, but they only displayed faint IR. There were lightly labeled small-sized cells in the molecular layer (ML), which appeared to represent basket cells. A few small-sized cells in the GCL were encountered, also exhibiting light intensity in general (Figure 8B). Sortilin IR was observed in all deep cerebellar nuclei apparently in association with neuronal somal profiles. In the dentate nucleus, the labeled cells showed light to moderate intensity, and were round, multipolar or irregular in shape, with neuronal processes representing dendritic elements identifiable on and between the labeled somata (Figure 8C,D).

#### Pons and Medulla Oblongata

In transverse sections of the pons at the level passing the upper part of the forth ventricle (4V), sortilin labeled cells were mostly impressive in the locus coeruleus (LC) located at the ventrolateral part of the ventricular floor (Figure 8E). The cells exhibited strong reactivity, were mostly round or oval in shape, and had short processes connected to the somata (Figure 8F, insert). Lightly stained cells were present in the PAG, and the dorsal raphe nucleus (DR) at the midline of ventricular floor. Additionally labeled cells were sparsely seen in other areas of the dorsal areas of the pons corresponding the RF. Labeled cells were rarely seen in the large part of the ventral and lateral areas of the pons occupied by the longitudinal and transverse neural fiber systems, including the central tegmental tract (CTT), median longitudinal fasciculus (MLF), superior cerebellar peduncle (SCP), lateral lemniscus (LL), spinal lemniscus (SL), medial lemniscus (ML), pontocerebellar tract (PCT) and the corticobulbar and corticospinal tracts (CBT, CST) (Figure 8E).

**Figure 9:**
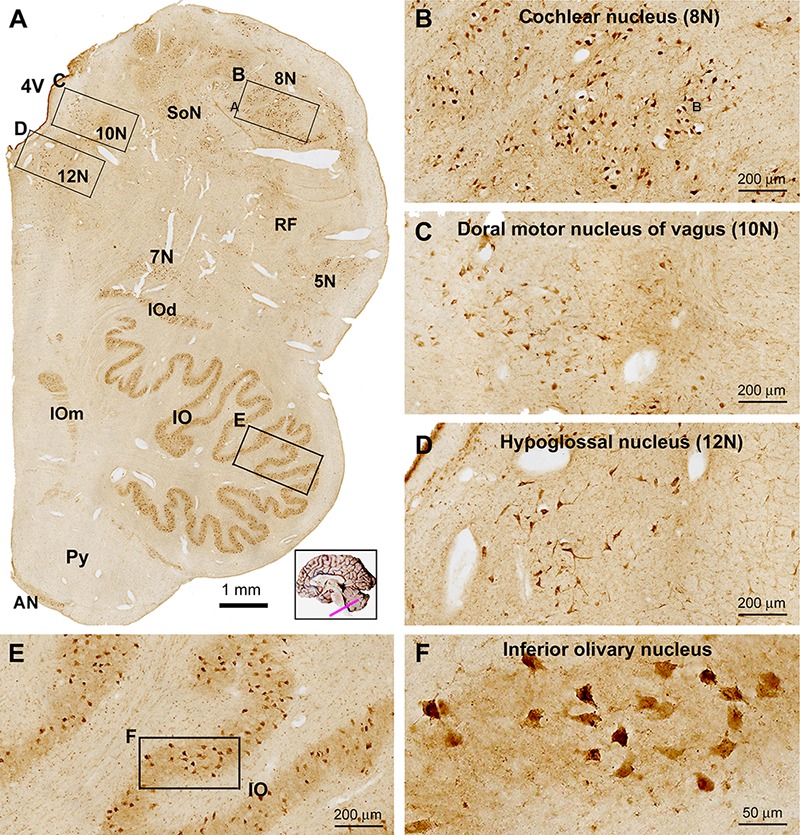
Low **(A)** and high **(B–F)** power views of sortilin IR in the adult human medulla oblongata (MO). At low magnification, labeled elements are mainly arranged as groups of cells located medial and lateral to the fourth ventricle (4V), and in the ventrolateral areas of the pons corresponding to the main and accessory nuclei (dorsal and medial, IOd and IOm) of the inferior olive (IO). Thus, labeled neurons are located in all cranial nerve nuclei at this level, including the cochlear and vestibular nuclei (8N), dorsal motor nucleus of vagus (10N), hypoglossal nucleus (12N), facial nucleus (7N), trigeminal sensory nucleus (5N), the solitary tract nucleus (SoN) and the reticular formation (RF) **(B–D)**. Neurons in the arcuate nucleus (AN) are also labeled, whereas the pyramid (Py) is unlabeled **(A)**. Labeled neurons in the IO are mostly multipolar in shape by closer examination **(E,F)**. Scale bars are as indicated in the panels.

In transverse sections of the medulla oblongata (MO) at the levels of the inferior olivary (IO) complex, groups of labeled cells corresponding to the cranial nerve nuclei were visualized ventral and lateral to the 4V, while the main and accessory nuclei of the IO were clearly marked in the ventral part of the oblongata (Figure 9A). Thus, the facial nucleus (7N), cochlear and vestibular nuclear complex (8N), nucleus of the solitary tract (SoN), sensory trigeminal nuclei (5V), dorsal nucleus of vagus (10N) and hypoglossal nucleus (12N) were readily distinguishable in the immunolabeled sections at low magnification. By closer examination the labeled cells in these nuclei were round/oval, multipolar and irregular in shape, and they possessed short or fairly long dendritic processes (Figure 9B–D). Labeled cells in the main IO nucleus as well as the accessory dorsal and medial IO nuclei (IOd, IOm) were mostly multipolar with short dendritic processes on the somata (Figure 9A,E). No labeling was seen in the cavity or hilus of the IO nuclei, suggestive of a lack of labeling to the axons leaving from the IO nuclei (Figure 9E,F). The pyramidal tract (Py) occupying the most medioventral part of the oblongata was essentially avoid of immunolabeling. There existed a small group of labeled cells around the medioventral surface of the pyramid, representing the neurons of the arcuate nucleus (AN) (Figure 9A).

### Quantification of Sortilin Labeling in Amygdalar and Cortical Neurons

**Figure 10:**
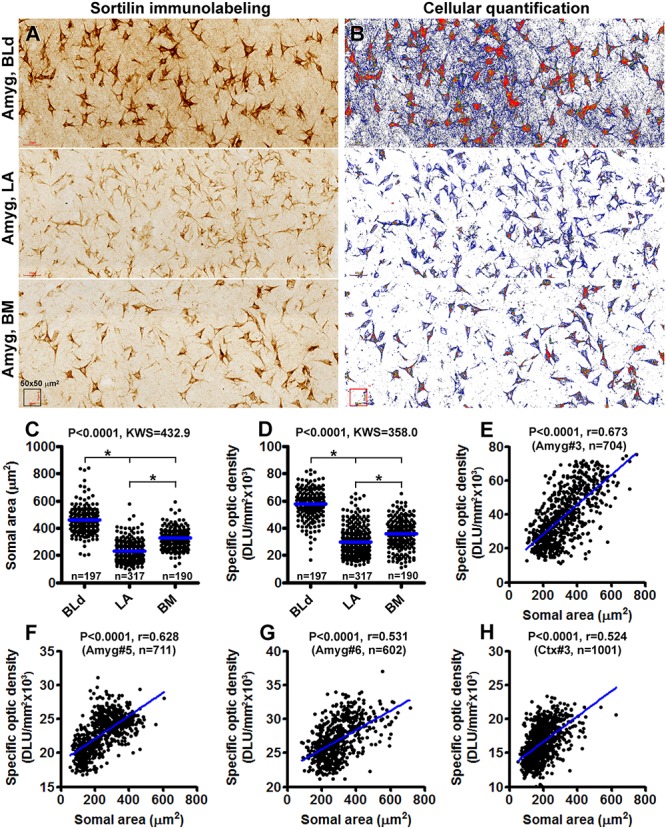
Correlative analysis on somal area and immunoreactivity of sortilin-labeled neurons in the basolateral dorsal nucleus (BLd), lateral nucleus (LA) and basomedial nucleus (BM) of the amygdaloid complex **(A–G)**, and the temporal neocortex **(H)**. Panel **(A)** is a combined image consisted of microscopic fields from the above nuclei as indicated, obtained from the same Motic-scanned image. Panel **(B)** is a screen-print working document viewed on the OptiQuant interface, which is in gray-scale TIFF format with pseudocolor representation of optic density variability among labeled profiles. Tracing marks surrounding individual neurons are visible, with somal area and total optic density, expressed as digital light units per square millimeter (DLU/mm^2^), reported for each circled neurons. Specific optic densities are calculated by using the optic density measured over a section processed in parallel but omitting the primary antibody incubation as a cut-off threshold. Panel **(C)** plots the somal areas of 197 neurons in the BLd nucleus, 317 neurons in the LA and 190 neurons in the BM of human brain #3. Panel **(D)** plots the specific optic densities of corresponding groups of individual neurons. The medians are significantly different between individual paring groups. Panel **(E)** is a correlation analysis for all measured neurons from the three nuclei, indicating a positive correlation (*p* < 0.0001, *r* = 0.673) between the somal area and labeling intensity among individual cells. Panels **(F,G)** plot the results of correlation analyses of amygdalar neurons measured in sections from brains #5 (*p* < 0.0001, *r* = 0.628) and brain #6 (*p* < 0.0001, *r* = 0.531). Panel **(H)** shows the positive correlation (*p* < 0.0001, *r* = 0.524) between somal size and labeling intensity of neurons measured over layers II–VI of the temporal neocortex from brain #3. Statistics reported by the non-parametric Kruskal–Wallis test with Dunn’s multiple comparison of medians **(C,D)** and Pearson correlation **(E)** are as indicated, (^∗^) with star signs indicating existence of significant intergroup difference. The numbers (n) of neurons measured are also labeled in the graph panels.

On examination of sections from different anatomical locations, it was impressive that neurons exhibiting the strongest sortilin IR were often the largest in size relative to other labeled cells in the same region. For examples, the large-sized pyramidal neurons in the neocortical layers III and V were heavily labeled, as were the large spiny neurons in both the neo- and paleo-striatum. This led us to speculate if there exists a correlation between somal size and sortilin expression among morphologically similar neurons or even different neurons. Therefore, we carried out quantitative cellular analyses over the subnuclei of the amygdaloid complex, included the BLd, LA and BM subdivisions, as well as overall layers II–VI of the temporal neocortex from 3 brains (case# 3, 5, and 6). Photographic regions (at 20× magnification) of the BLd, LA, and BM, and cortical layers II–VI, were obtained from the same Motic-scanned image and incorporated into a single file (Figure 10A), which was subsequently converted into gray-scale format, followed by simultaneous measurements of somal areas and total optic densities of individual neurons (Figure 10B). Specific optic densities, expressed as digital light units per square millimeter (DLU/mm^2^), of labeled neurons were calculated by using the optic density (background) measured from batch-processed sections without the exposure to the primary antibody as a cut-off threshold.

A total of 197 sortilin labeled neurons in the BLd nucleus, 317 neurons in the LA and 190 neurons in the BM were measured for brain case #3. The mean somal area was significantly larger for the neurons in the BLd (462.0 ± 99.7 μm^2^) relative to the LA (224.7 ± 58.7 μm^2^) and the BM (324.5 ± 76.8 μm^2^), with statistically significant difference in the medians between the 3 individual nuclear groups [*P* < 0.0001, Kruskal–Wallis statistic (KWS) = 432.9] (Figure 10C). The mean specific optic density was also significantly greater for the neurons in the BLd (57826 ± 10868 DLU/mm^2^) relative to LA (29789 ± 10341 DLU/mm^2^) and BM (35671 ± 10937 DLU/mm2), with statistical difference in the medians between the 3 individual nuclear groups (*P* < 0.0001, KWS = 358.0) (Figure 10D). We further plotted somal sizes relative specific optic densities of individually measured neurons from all the three nuclei, which revealed a positive correlation between the two indices in the entire neuronal population (*P* < 0.0001, *r* = 0.673) (Figure 10E). Measurements from the brains cases # 5 and #6 revealed similar differences as the above in regard to the somal size and labeling intensity of neurons between the amygdaloid subnuclei (graphs not shown), with a positive correlation between the two indices while the measurements from individual neurons were plotted together (Figure 10F,G). We carried out the same type of cellular quantification as above over cortical layers II–VI of the temporal neocortex (also using sections passing the amygdaloid complex) from brain case #3, 5 and 6. Shown as an example (case #3), there was also a positive correlation between the somal size and intensity of sortilin immunolabeling among the populations of neocortical neurons (Figure 10H).

### Immunofluorescent Characterization of Sortilin-Labeled Cells

**Figure 11:**
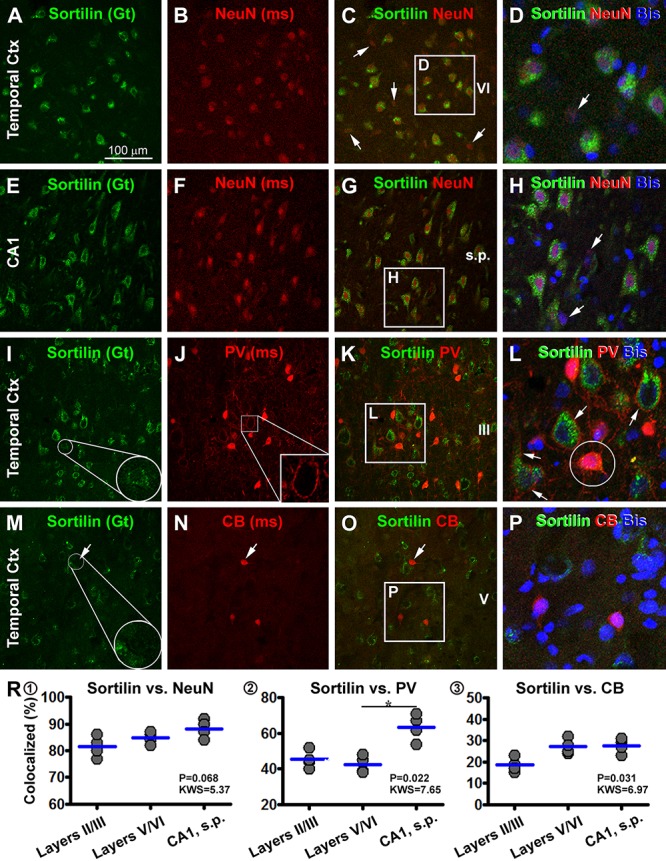
Confocal double immunofluorescent characterization of sortilin labeling with neuronal markers. Antibody markers and imaged areas are as indicated, with cell nuclei displayed by bisbenzimide (Bis) stain in blue. Panels **(A–H)** show a frequent colocalization of sortilin with the neuronal nuclear antigen (NeuN) in the temporal cortex (Ctx), although some NeuN+ cells (arrows) exhibits little sortilin IR. Panels **(I–L)** show that parvalbumin (PV)+ interneurons exhibit infrequent sortilin colocalization. However, a sortilin+ granules can be found in the PV+ somata (enlarged round inserts) **(I,L)**. At high magnification PV+ terminals occur surrounding the somata and dendrites of pyramidal cells with bright sortilin IR **(J,L)**. Panels **(M–P)** show a lack of clear sortilin labeling in calbindin (CB)+ interneurons. Panels **(R1–3)** plot quantification of the colocalization rates. Clear sortilin labeling is seen in over 80% NeuN+ neurons (R1), whereas sortilin labeling (weak intensity or granular elements) is seen in ∼40–60% PV+ neurons (R2), and in ∼20–30% CB+ neurons (R3). ^∗^*p* < 0.05 by intergroup comparison. Scale bar = 100 μm in **(A)** applying to **(B,C,E–G,I–K,M–O)**, equivalent to 33 μm for the remaining panels.

We carried out multiple sets of double immunofluorescent characterization to determine the cellular phenotypes of sortilin-labeled cells in selected brain structures, including the cerebral neocortex, hippocampal formation, cerebellar cortex and several subcortical locations. As assessed in sections of temporal neocortex and hippocampal formation, sortilin IR appeared to mostly colocalize with NeuN, the common mature neuronal marker (Figure 11A–H). An effort was taken to quantify the rate of colocalization between sortilin and NeuN, by cell counting over 20× confocal images of non-overlapped (alternatively spaced in one microscopic field apart) fields along layer III (covering layer III and part of layer II) and along layers V/VI of the middle temporal gyrus, and along the CA1 sector of the hippocampus (20 fields/brain). For each of the above regions, About 100 NeuN immunofluorescent cells per case (used cases #3, 4, 5, and 6) were analyzed, with those displayed distinct sortilin colabeling counted. Among the total amount of NeuN-labeled neurons, 81.5 ± 3.9% cells (mean ± SD, same format below) in cortical layers II/III, 84.8 ± 2.2% in layers V/VI and 88.3 ± 3.5% in CA1 were seen to exhibit distinct sortilin labeling, while there was no region-related difference in the colocalization rate (*F* = 0.068, KWS = 5.37) (Figure 11R1).

We also examined sortilin colocalization with markers of GABAergic inhibitory interneurons with double immunofluorescence. In double labeling for sortilin with Sp8, a putative common marker of cortical GABAergic interneurons, we did not find an impressive colocalization of the two markers (data not shown). We further analyzed sortilin colabeling with PV and CB, two excellent markers for GABAergic subpopulations (Yan et al., 1996). By overall scanning of the merged images, sortilin appeared to visualize largely a separate population of cells different from that of PV and CB labeled ones in the same microscopic field (Figure 11I–P). The somata and proximal dendrites of sortilin immunoreactive neurons were surrounded by PV immunoreactive terminals (Figure 11I–L), characteristic of GABAergic perineuronal nets around excitatory cortical pyramidal neurons (Enwright et al., 2016). However, by closer examination some PV or CB labeled interneurons contained sortilin IR as intracellular granules or along the somal surface (Figure 11I,M). We carried out cell count in the same manner as above for the sortilin/NeuN analysis. Among the total PV-labeled interneurons (based on ∼100 neurons counted per brain for a given region), 45.3 ± 4.9% of them in cortical layers II/III, 42.5 ± 4.8% in layers V/VI and 63.5 ± 7.3% in CA1, were seen to exhibit weak sortilin IR on the membrane or in the cytoplasm. There was an overall difference between the medians (*F* = 0.022, MWS = 7.65), with *post hoc* tests (Dunn’s Multiple Comparison Test) indicated a higher rate in CA1 than layers V/VI (Figure 11R2). Among the total CB-labeled interneurons (based on 200–300 neurons counted per region per brain), 18.5 ± 3.4% in cortical layers II/III, 27.3 ± 3.6% in layers V/VI and 27.5 ± 3.4% in CA1, exhibited weak but visible sortilin IR, while there was no difference between the measured individual regions by *post hoc* test (*F* = 0.031, MWS = 6.91) (Figure 11R3).

**Figure 12:**
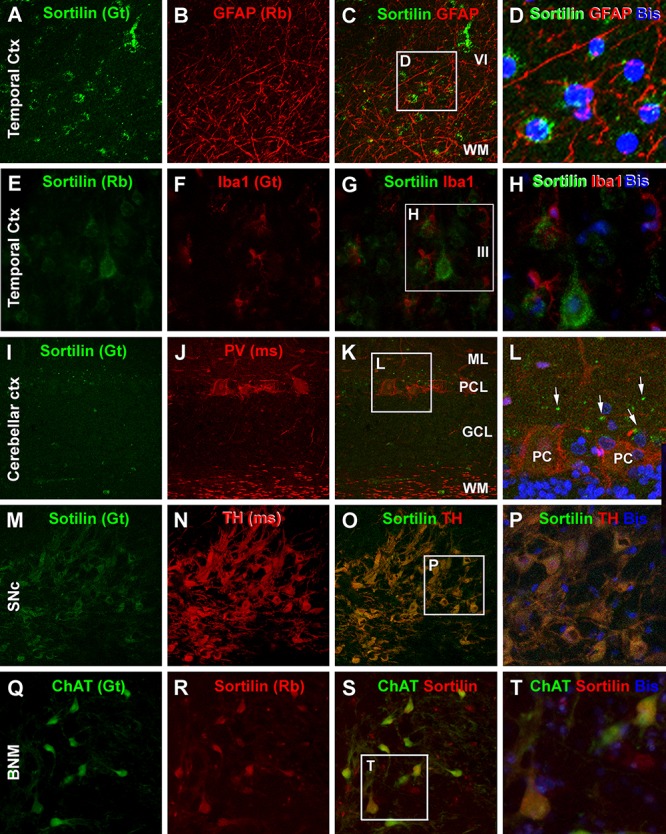
Confocal double immunofluorescent characterization of sortilin labeling relative to glial and additional neuronal markers. Antibody markers and imaged areas are as indicated, with cell nuclei displayed by bisbenzimide (Bis). Panel **(A–D)** show that sortilin labeling does not colocalize with immunofluorescence of glial fibrillary acidic protein (GFAP), an astrocytic marker, around the border of layer VI and the white matter (WM) of the temporal cortex (Ctx). There is also no colocalization between sortilin and Iba1, a marker of microglia **(E–H)**. Panels **(I–L)** show a lack of sortilin labeling in cerebellar Purkinje cells (PC) displaying distinct PV immunoreactivity in the somata and in axons in the WM. Sortilin labeled dot-like elements (white arrows) are present around the Purkinje cell somata. Panels **(M–P)** demonstrate a complete colocalization of sortilin in dopaminergic neurons in the pars compacta of the substantia nigra (SNc) as visualized by the tyrosine hydroxylase (TH) antibody. Panels **(Q–T)** illustrate the colabeling of sortilin and choline acetyltransferase (ChAT) in a population of large-sized neurons in the basal nucleus of Meynert (BNM). ML, molecular layer; PCL, Purkinje cell layer; GCL, granule cell layer. Scale bar = 100 μm in **(A)** applying to **(B,C,E–G,I–K,M–O,Q–S)**, equivalent to 33 μm for the right-side high magnification panels.

In contrast to the neuronal markers, sortilin IR in the temporal neocortex was not found to colocalize with immunofluorescence of GFAP, the commonly used astrocytic marker (Figure 12A–D). There was also no any immunofluorescent colocalization of sortilin with Iba1, a microglial marker (Figure 12E–H). Sortilin with PV and CB (not shown) double immunofluorescence was also carried in cerebellar cortical sections. Sortilin IR was faint or not readily detectable in the somata and dendrites of Purkinje cells (Figure 12I), While PV immunofluorescence was distinct in somata, dendrites as well as axonal processes of Purkinje cells (Figure 12J,K). Consistent with the peroxidase-DAB labeling, small dot-like sortilin immunoreactive elements were seen around the Purkinje cell layer between the somata and proximal dendrites of the Purkinje cells (Figure 12L). In addition to the above, we carried out confocal double immunofluorescence in several subcortical structures including the BNM, SNc, LC and brainstem. Sortilin and TH colocalization was invariably seen among neurons in the SNc (Figure 12M–P) as well as the LC (not shown), indicating sortilin expression in dopaminergic neurons and noradrenergic neurons, respectively. A complete colocalization of sortilin in ChAT positive multipolar cells was detected in sections covering the basal forebrain region over the area of BNM (Figure 12Q–T). For these later sets of double labeling characterizations, we did not quantify the colocalization rates because based on examination of sections from different brains, a lack of colocalization (sortilin relative to GFAP and Iba1 in cerebral neocortex, sortilin relative to PV/CB in cerebellar Purkinje cells) or a complete colocalization (sortilin relative to TH in SNc and LC, and sortilin relative to ChAT in BNM) was consistently observed.

## Discussion

### Human Brain Sortilin Expression in Comparison With Existing Rodent Data

The present study shows a widespread sortilin IR in the human brain across major anatomical locations, with considerable laminar and neuronal phenotype variability. The findings can be summarized as the following (Table 2). (1) Sortilin IR occurs in all forebrain, midbrain and hindbrain-derived structures in the adult human brain, as characterized in the OB, cerebrum, basal ganglia, thalamus, midbrain, brainstem and cerebellum; (2) Sortilin IR appears to be preferentially associated with neuronal cells but not or less so with glial cells, based on morphological criteria and as confirmed by its colocalization with NeuN but not with GFAP or Iba1; (3) Among neurons, sortilin IR appears to more distinctly present in the principal/projective neurons, as judged per morphological terms and an infrequent sortilin colocalization with interneuron markers in the cerebral neocortex. DAB-based immunolabeling also shows that several interneuron populations in subcortical structures, e.g., the small spiny neurons in the striatum and basket cells in the cerebellar cortex exhibit light sortilin IR; (4) Sortilin IR is commonly associated with neurons at the relay centers on most neuroanatomical pathways in brainstem, including the dopaminergic, noradrenergic and cholinergic neurons that project to and modulate cerebral functions; (6) In the same brain region, neurons with the largest somal size are often seen to show the heaviest sortilin IR, except for the mitral cells in the OB and Purkinje cells in the cerebellar cortex, which are the largest cells in the corresponding regions; (8) Finally, it should be noted that in the cerebellar cortex, some granular elements around the PCL show fairly intense sortilin IR, while their precise identity remains to be determined.

**Table 2:** Semi-quantification of sortilin immunoreactivity in major neuroanatomical regions and neuronal populations in the adult human brain.

Structure and neuronal types	Immunolabeling intensity	Specific note
**Olfactory bulb**		
Periglomerular neurons	(-)	
Mitral and tufted cells	(-)	
Granule cells	(-)	Subependymal cells (+)
**Cerebral neocortex**		
Layer I	(-/+)	Large pyramidal neurons in layers III and V (++++); interneurons (+/++)
Layer II/III	(+++)	
Layer IV	(+/++)	
Layer V	(+++)	
Layer VI	(+++)	
White matter	(-)	
**Amygdala and basal forebrain**		
Central nucleus	(+++)	
Basolateral nucleus	(+++)	
Lateral nucleus	(++)	
Basomedial nucleus	(++/+++)	
Basal nucleus of Meynert	(+++)	Sortilin and ChAT colocalization observed
**Hippocampal formation**		
Entorhinal cortex	(+++)	
Prosubiculum	(+++)	
Subiculum	(+++)	
Presubiculum	(+++)	
Parasubiculum	(+++)	
CA1-CA3 (pyramidal neurons)	(+++)	Thorny excrescences on CA3 and mossy cells (+++); interneurons (+/++)
Hilus (mossy cells)	(+++)	
Granule cells	(++++)	
**Basal ganglia**		
Caudate	(++)	Large spiny ChAT neurons (++++); small spiny neurons (++)
Putamen	(+)	
Globus pallidus	(++)	
Claustrum	(+++)	
**Diencephalon**		
Thalamus		
anterior nuclear group	(++)	
medial nuclear group	(++)	
posterior nuclear group	(++)	
lateral geniculate nucleus	(++)	
medial geniculate nucleus	(++)	
Hypothalamus	(++)	
**Mesenphalon (midbrain)**		
Substantia nigra pars compacta	(+++)	Sortilin/TH colocalization observed
Substantia nigra pars reticulata	(-/+)	
Ventral tegmentum area	(++)	
Red nucleus	(++)	
Periaqueductal gray	(++)	
Oculomotor nucleus (CN3)	(++)	
Trochlear nucleus (CN4)	(++)	
Superior colliculus	(++/+++)	Large multipolar cells in the deep layers (+++)
Inferior colliculus	(++/+++)	
**Pons**		
Locus coeruleus	(++++)	Sortilin/TH colocalization observed
Motor trigeminal nucleus (CN5)	(+++)	
Sensory trigeminal nucleus (CN5)	(++)	
Median raphe nucleus	(+++)	
Pontine nucleus	(++)	
Superior olivary nuclei	(++)	
**Medulla oblongata**		
Vestibular nuclei (CN8)	(+++)	
Cochlear nuclei (CN8)	(+++)	
Inferior olivary nuclei	(++)	
Nucleus of the solitary tract	(+++)	
Spinal trigeminal nucleus (CN5)	(+++)	
Abducent nucleus (CN6)	(+++)	
Facial nucleus (CN7)	(+++)	
Dorsal motor nucleus vagus (CNX)	(+++)	
Hypoglossal nucleus (CN12)	(+++)	
**Cerebellum**		
Molecular layer (basket cells)	(+/++)	
Purkinje cell layer (Purkinje cells)	(-/+)	Extracellular granular profiles (++)
Granule cell layer (granule cells)	(-/+)	
White matter	(-)	

*(-), background level as seen in the white matter; (+), weak labeling with cell outline visible; (++), moderate labeling with cell body and dendritic processes well visualized; (+++), strong labeling with somata and dendritic processes distinctly visualized; (++++), strongest labeling with somata and dendrites heavily stained; CN, cranial nerve nucleus; ChAT, choline acetyltransferase; TH, tyrosine hydroxylase*.

Data regarding the whole brain expression pattern of sortilin in mammalian species are limited to date. An early study, which used a different antibody from the ones used here, reported the distribution of sortilin IR in the rat central nervous system (Sarret et al., 2003a), with several other studies described the neuronal expression of sortilin in selected regions in rodent and human brains, primarily the cerebral cortex and hippocampal formation (Gutekunst et al., 2003; Al-Shawi et al., 2008; Mufson et al., 2010; Hu et al., 2017; Zhou F.Q. et al., 2018). According to the rat study (Sarret et al., 2003a), sortilin IR is widely distributed in the brain. Thus, the labeling in the cerebral neocortex and paleocortex is mostly distinct in layers II and V pyramidal neurons. In the hippocampal formation, labeled neurons appear to be mainly interneuron-like and are distributed in the s.p. as well as s.o. and s.r., whereas rare labeling is present in the dentate gyrus. In the cerebellar cortex, the somata and dendrites of Purkinje cells exhibit heavy labeling, whereas other cellular elements show little labeling. Thus, there appear to have substantial difference in the regional and cellular expression of sortilin in rodent brain relative to humans as revealed in the current study. Notably, in a recent publication used the same goat antibody as in the current study (Boggild et al., 2018), sortilin IR is found to be highly expressed in embryonic telencephalon of rats, with the cortical plate and the developing allocortex exhibited strongly labeled neuronal perikarya and dendrite-like processes. In comparison, sortilin IR is much limited in more caudal brain levels including the cerebellum (Boggild et al., 2018). Another recent study also shows a greater sortilin expression in the forebrain than hindbrain in mice, with the cerebellum displayed fairly low labeling (Johnson et al., 2017).

### Differential Sortilin Expression Among Neuronal Populations

Our current mapping data indicate that, in general, relatively large-sized principal/projective neurons show microscopically more evident sortilin expression as compared to interneurons in multiple brain regions. The morphometric analysis establishes that among amygdalar and neocortical neurons, those in larger somal size tend to have stronger sortilin expression. The relatively large-sized PV neurons contain granule-like sortilin-IR, with a higher sortilin colabeling seen in those in CA1 relative to the neocortex, likely because hippocampal PV neurons are often relatively large while cortical PV neurons are more variable in size. Sortilin expression is generally low or minimal in CB cortical neurons that are much smaller than principal neurons (Yan et al., 1996; Cao et al., 1997). However, it should be noted that cerebellar Purkinje cells and OB mitral cells are large-sized neurons in the corresponding regions, and are GABAergic and glutamatergic, respectively, while neither exhibits strong sortilin IR. The GABAergic nature of Purkinje cells might be a reason for their lack of impressive sortilin expression. Purkinje cells and mitral cells also appear quite unique relative to other neurons as they do not express the common neuronal marker, NeuN (Herculano-Houzel and Lent, 2005). It is of interest to note that a recent study shows segregated localizations of sortilin and SorCS2, another member of the vps10p family, in mouse brain during embryonic to postnatal development. Specifically, SorCS2 rather than sortilin is highly expressed in the cerebellum and OB (Boggild et al., 2018). Thus, the regionally segregated expression of vps10p family proteins might also account for a low expression of sortilin among the neurons in the cerebellum and OB.

Somal size and shape of neurons are two basic morphological parameters closely related to the territory/orientation of dendritic arborization and axonal projection that determine neuronal network organization and signal transmission (Herculano-Houzel et al., 2015). The observation of somal size as a factor correlating to the amount of sortilin expression might be of functional relevance. Recent cell biology and *in vivo* studies have increasingly recognized the role of sortilin for protein sorting, trafficking and homeostasis in neurons (Allard et al., 2018; Hirst et al., 2018; Itoh et al., 2018; Paushter et al., 2018; Yabe-Wada et al., 2018). Neurons are unique relative to other bodily cells most prominently as they generate and transmit electronic impulses to communicate between each other. This physiological property is carried out by structurally specialized cellular apparatus, dendrites and axons, which can extend far away from their parent somata. Larger somal size means a greater cell surface for ligand-receptor interaction as well as the operation and maintenance of membrane-associated molecular systems responsible for signaling functions. Larger neurons may have a greater load of the operation and maintenance of protein sorting/trafficking along lipid membrane and between intracellular compartments. Thus, based on its assumed role in transmembrane signaling and intracellular protein trafficking in neurons, one can expect that larger neurons may require a higher supply of sortilin to carry out the above cellular functions. Further, because sortilin is primarily expressed in the somata, dendritic processes and spines, this VPS10P member appears to mainly support protein sorting in the somatodendritic, including postsynaptic, compartments, but may not play a significant role in axonal protein transportation.

### Implications for Pharmacological Intervention on Central Sortilin Function

As denoted in the Introduction, NT has been considered a drug target for some neurological and psychiatric conditions, with efforts being taken to develop pharmaceutically usable agonists and antagonists to its receptors, including sortilin as NTR3 (Lazarus et al., 1977; Herve et al., 1986; Köhler et al., 1987; Betancur et al., 1998; Hermans and Maloteaux, 1998; Lahti et al., 1998; Quistgaard et al., 2009; Andersen et al., 2014). In fact, sortilin has a fairly high affinity (Kd = 0.1–0.3 nM) to NT, comparable to that of NTR1 (Alexander and Leeman, 1998), and thus could be sensitively affected by NTR3 agonization or antagonization. More recent studies show sortilin involvement in many neurobiological and neuropathological events, such as regulation of neuronal/synaptic viability and activity via its binding and trafficking of neurotrophic factors, mood disorders, dementia and AD-type neuropathology (Hu et al., 2010; Nykjaer and Willnow, 2012; Capsoni et al., 2013; Johnson et al., 2017; Ruan et al., 2018; Xu et al., 2018). This has also raised discussion of pharmacological manipulation of sortilin as a disease modifying strategy, regardless whether it involves neurotensin signaling.

The current mapping study indicates that sortilin is enriched in many neuronal populations of the human brain, such as the cerebral principal neurons as well as subcortical dopaminergic, noradrenergic and cholinergic neurons. The anatomical data appear to support the notion that pharmacological modulation of sortilin, as NTR3, may influence the dopaminergic, noradrenergic and cholinergic neurotransmissions, which has been proposed as therapeutic options for Parkinson’s disease, schizophrenia and affective disorders (Gully et al., 1993; Kitabgi, 2002; Kinkead and Nemeroff, 2006; Ferraro et al., 2009; McGonigle, 2012). However, the broad expression of sortilin in multiple neuronal populations as seen in the human brain also raises a concern of substantial neurological side-effects. Therefore, in future development and evaluation of centrally and peripherally acting drugs for some specific diseases or clinical conditions (Baxendale et al., 2013; Kleczkowska and Lipkowski, 2013; Ouyang et al., 2017), cautions must bee taken to weigh the wanted and untoward effects likely arising from off- or cross-target blocking or activation of sortilin activity in different neuronal populations or neuroanatomical systems.

In summary, the present study has extended a regional and cellular mapping of sortilin expression in the adult human brain through immunohistochemical characterization. Sortilin immunolabeling occurs broadly in cortical and subcortical structures, with large-sized neurons exhibiting heavier labeling relative to smaller ones. Principal/projective neurons including pyramidal, multipolar and polymorphic neurons in the cerebral cortex, hippocampal formation and amygdala, as well as subcortical dopaminergic, noradrenergic and cholinergic neurons, are among the neurons with enriched sortilin expression.

## Author Contributions

S-YX, Q-LZ, QZ, LW, JJ, and TT contributed to tissue processing, immunohistochemistry, and data collection. QZ, Q-LZ, AP, and X-XY contributed to brain banking. JM and X-XY contributed to neuropathological evaluation. S-YX, Q-LZ, TT, and YC contributed to image capture and analysis. X-XY contributed to funding acquisition, experimental design and manuscript composition.

## Conflict of Interest Statement

The authors declare that the research was conducted in the absence of any commercial or financial relationships that could be construed as a potential conflict of interest.
