# Supplementary material for: Regional and Cellular Mapping of Sortilin Immunoreactivity in Adult Human Brain
Source: Front Neuroanat. 2019 Mar 12;13:31. doi: 10.3389/fnana.2019.00031 (PMC6422922; doi:10.3389/fnana.2019.00031)

Supplemental Figure 1: Method for blocking tissue samples from the formalin-fixed half brain for histological processing. Framed areas are individual brain regions or structures to be blocked from 1 cm thick slices orderly prepared from the frontal to occipital poles of the cerebrum, as labeled.

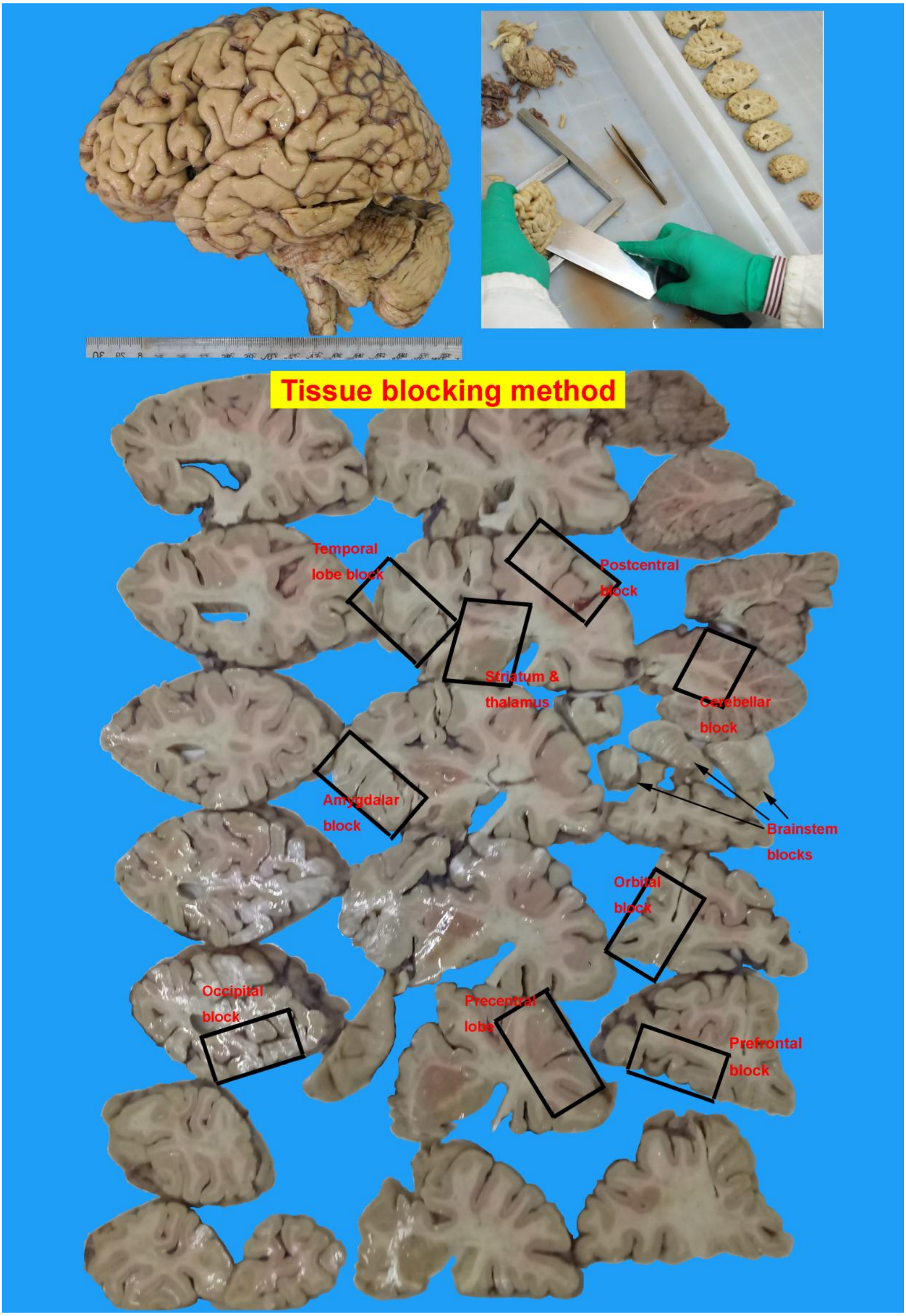

Supplemental Figure 2: Verification of the specificity of the goat (AF3154) and rabbit (ab16640) anti-sortilin antibodies in human prefrontal and cerebellar cortical sections. The left panels show labelings in the absence of competing antigenic peptides. The middle panels show diminished labelings in the presence of the corresponding competing peptides at 5 and 10 times of the concentration of the primary antibodies, respectively. The right panels show the lack of labeling while the staining is processed with the primary antibody omitted in the incubation buffer.

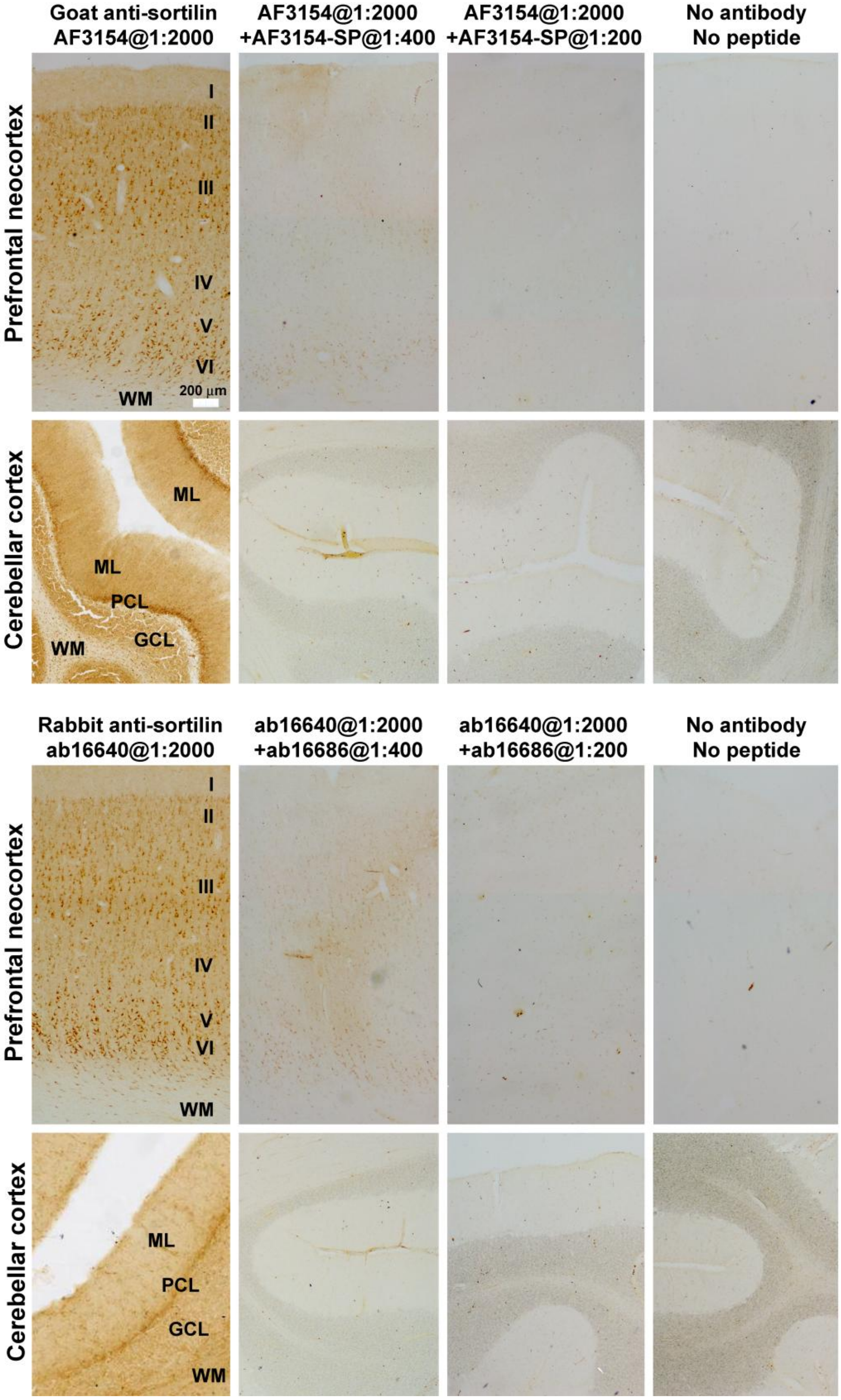

Supplement: Supplementary file 1 [file Data_Sheet_1.PDF]
